# Depression-Associated Gene *Negr1-Fgfr2* Pathway Is Altered by Antidepressant Treatment

**DOI:** 10.3390/cells9081818

**Published:** 2020-07-31

**Authors:** Lucia Carboni, Francesca Pischedda, Giovanni Piccoli, Mario Lauria, Laura Musazzi, Maurizio Popoli, Aleksander A. Mathé, Enrico Domenici

**Affiliations:** 1 Department of Pharmacy and Biotechnology, Alma Mater Studiorum Università di Bologna, 40126 Bologna, Italy; 2 Department of Cellular, Computational and Integrative Biology, University of Trento, 38123 Trento, Italy; francesca.pischedda@unitn.it (F.P.); giovanni.piccoli@unitn.it (G.P.); enrico.domenici@unitn.it (E.D.); 3 Department of Mathematics, University of Trento, 38123 Trento, Italy; lauria@cosbi.eu; 4 Fondazione The Microsoft Research—University of Trento Centre for Computational and Systems Biology (COSBI), 38068 Rovereto (Trento), Italy; 5 School of Medicine and Surgery, University of Milano-Bicocca, 20900 Monza, Italy; laura.musazzi@unimib.it; 6 Laboratory of Neuropsychopharmacology and Functional Neurogenomics, Dipartimento di Scienze Farmaceutiche, Università degli Studi di Milano, 20133 Milano, Italy; maurizio.popoli@unimi.it; 7 Karolinska Institutet, Department of Clinical Neuroscience, SE-11221 Stockholm, Sweden; aleksander.mathe@ki.se

**Keywords:** major depressive disorder, antidepressants, cell adhesion molecules, rodent models, Flinders sensitive line

## Abstract

The *Negr1* gene has been significantly associated with major depression in genetic studies. *Negr1* encodes for a cell adhesion molecule cleaved by the protease Adam10, thus activating Fgfr2 and promoting neuronal spine plasticity. We investigated whether antidepressants modulate the expression of genes belonging to *Negr1-Fgfr2* pathway in Flinders sensitive line (FSL) rats, in a corticosterone-treated mouse model of depression, and in mouse primary neurons. *Negr1* and *Adam10* were the genes mostly affected by antidepressant treatment, and in opposite directions. *Negr1* was down-regulated by escitalopram in the hypothalamus of FSL rats, by fluoxetine in the hippocampal dentate gyrus of corticosterone-treated mice, and by nortriptyline in hippocampal primary neurons. *Adam10* mRNA was increased by nortriptyline administration in the hypothalamus, by escitalopram in the hippocampus of FSL rats, and by fluoxetine in mouse dorsal dentate gyrus. Similarly, nortriptyline increased *Adam10* expression in hippocampal cultures. *Fgfr2* expression was increased by nortriptyline in the hypothalamus of FSL rats and in hippocampal neurons. *Lsamp*, another IgLON family protein, increased in mouse dentate gyrus after fluoxetine treatment. These findings suggest that *Negr1-Fgfr2* pathway plays a role in the modulation of synaptic plasticity induced by antidepressant treatment to promote therapeutic efficacy by rearranging connectivity in corticolimbic circuits impaired in depression.

## 1. Introduction

Recent genome-wide association studies (GWAS) have identified *Negr1* (neuronal growth regulator 1) as one of the most significant risk genes for major depressive disorder (MDD) [1,2], and by integrating GWAS with gene expression information across multiple human tissues, including brain, the association has been confirmed [3]. The regulatory mechanisms by which polymorphism in *Negr1* may confer MDD risk have been recently investigated in silico [4] and in vitro [5] pointing to the identification of putative causal variants for MDD. The involvement of *Negr1* in the molecular neurobiology of MDD is also suggested by reports showing changes in protein expression patterns in cerebrospinal fluid of depressed patients [6], and in response to antidepressant treatments [7]. In addition, a contribution to Alzheimer’s disease risk has also been reported, supporting the relevant role of *Negr1* in neuronal health and disease [8,9].

Intriguingly, different polymorphisms in the *Negr1* locus are also known to be associated with risk for body mass index and obesity [10,11]. The biological mechanism is unknown, but available data suggest that *Negr1* effects on energy balance are mediated at least in part by genes in the rodent hypothalamus, particularly in the periventricular hypothalamic areas [12,13].

Efforts aimed at elucidating the molecular mechanisms responsible for the genetic associations examined the effect of gene modulation on behaviour in animal models. Available evidence suggests that *Negr1* gene deficiency impairs sociability and social dominance, and is able to induce learning deficits [14,15]. At the molecular level, Negr1 is a cell adhesion molecule belonging to the immunoglobulin superfamily. Within this family, IgLON sub-family members include Negr1 (also known as Kilon), Lsamp, OPCML, and neurotrimin; these highly glycosylated proteins display three immunoglobulin domains and are anchored to the cell membrane by a glycosylphosphatidylinositol motif inserted in their C termini [16]. Lsamp direct interaction with Negr1 has been previously observed in protein microarray experiments [17] and recently confirmed in mouse brain [18]. Lsamp function in serotonergic signalling has been suggested by the findings that *Lsamp*^–/–^ mice showed lower 5-HT levels and higher 5-HT turnover, which have been called to explain their low anxiety and aggression [19,20]. In addition, *Lsamp* genetic polymorphisms showed significant associations with MDD and schizophrenia [21,22], thus providing further support to the hypothesis that IgLON members are involved in the neurobiological basis of neuropsychiatric disorders.

*Negr1* is widely expressed in the adult central nervous system, with high levels in the hippocampus, frontal cortex, and hypothalamus [13,14,15,23], where it is implicated in white matter integrity [23]. Moreover, Negr1 promotes neurite growth, controlling the development of neurite arborisation and supporting axon connections [14,24], thus playing a critical role in neuronal development. Consistently, its deficiency during development results in brain abnormalities including ventricle enlargement and decreased brain volume [15].

Our previous studies clarified the biological role of a pathway involving Adam10, Negr1, and Fgfr2 in neurite tree outgrowth and maturation, suggesting a key role in neuronal structural development [25]. Intriguingly, decreased expression of *Fgfr2* in the dorsolateral prefrontal cortex (DLPFC) and in the anterior cingulate cortex were found in post-mortem brains in MDD patients [26].

Mood disorders are the major cause of “Years of life lived with disability” and “Years of life lost because of premature death” due to illness itself, somatic comorbidity, and suicide [27,28]. The problem is growing due to the increased life-span and higher MDD frequency with increasing age [29]. In spite of the wealth of data on the monoaminergic system and the hypothalamus-pituitary-adrenal axis, understanding of the disease pathophysiology remains limited [30]. Efficacious therapies are mainly based on the pharmacological treatment with antidepressants belonging to different classes, the high majority of which share the common feature of addressing the monoaminergic circuits [31]. Among them, the older tricyclic antidepressants mainly target the noradrenaline and serotonin transporters, whereas agents belonging to the serotonin-selective reuptake inhibitors specifically address serotonergic reuptake. Using currently available drugs in the clinic, 30–40% of depressed patients fail to respond adequately [32]. Consequently, there exists a major unmet need to develop more efficient treatments based on novel mechanisms of action.

The discovery of new targets for therapeutic intervention is facilitated by studying animal models displaying depressive-like behaviours and neurobiological abnormalities that resemble those observed in MDD patients. Among the animal species, the Flinders sensitive line rats (FSL) are a selected strain that phenotypically displays many depression symptoms, including increased immobility in the forced swim test, reduced activity in novel open-field behaviours, decreased appetite and weight loss, sleep disturbances, and neurochemical abnormalities, in particular decreased expression of neuropeptide Y, similar to those found in humans [33,34]. Since the exposure to early-life adversities is a major precipitating factor for MDD in predisposed individuals [35], the experience of post-natal maternal separation (MS) represents a widely used animal model of MDD in rodents which we have already characterised in FSL rats [36,37,38,39,40,41,42]. In mice, it has been demonstrated that long-term exposure to glucocorticoids induces anxiety and depressive-like states, which are amenable to be reversed by antidepressant treatments [43].

The aim of this study was to assess if different antidepressant treatments affected the expression of *Negr1* and other genes belonging to the same pathway in rodent depression models. Different antidepressants were investigated with the object of discovering whether a common response could be detected. Chronic treatments were administered to investigate long-term neurobiological adaptations which are supposed to mediate therapeutic efficacy [44]. The focus was on brain regions in which abnormality of corticolimbic connectivity may underlie MDD [45] and on the hypothalamus, since it regulates eating behaviour and neuro-vegetative symptoms of MDD. Further experiments were carried out on mouse neuronal primary neurons to examine whether molecular alterations were directly induced on cells by antidepressants.

## 2. Materials and Methods

### 2.1. Animals

The FSL rats derived from the colonies maintained at the Karolinska Institutet. Animal handling and experimental procedures were performed in accordance with the European Community Council Directive 86/609/EEC and were approved by Karolinska Institutet Animal Ethical Committee (Ethical approval N 408/10). All efforts were made to minimise animal distress and to reduce the numbers of animals used. FSL rats were housed in plastic cages at a temperature of 22 °C ± 1 °C and 45–55% humidity in a light and dark schedule of 12 h with lights on at 07:00 am and with food and water freely available.

### 2.2. Behavioural Procedures

MS was performed as previously reported [36]. The separation procedures were performed from post-natal day 2 to 14. At 09:00 am, pups belonging to the MS group were removed from the dam to a different cage in a separate room for 180 min, without dividing the litter. Rats belonging to the control group (n-MS) were left undisturbed except for routine cleaning of the cages. Pups were weaned on post-natal day 23, separated by sex, and 3 to 5 animals were housed per cage. Only male rats were included in the study.

### 2.3. Antidepressant Treatment in FSL Rats

Six-week old rats belonging to MS and n-MS groups were randomly split into three groups receiving escitalopram (ES), a serotonin-selective reuptake inhibitor, nortriptyline (NT), predominantly a noradrenaline reuptake inhibitor, or vehicle for one month. The antidepressant was administered in food chow (0.34 g/kg chow for 3 weeks, 0.41 g/kg chow during the rest of the experiment; the antidepressants were provided by H. Lundbeck A/S, Nykøbing Sjælland, Denmark). The antidepressant dose corresponded approximately to 25 mg/kg body weight/d, in agreement with previous results [40,41,42,46]. Experimental groups consisted of 6 rats. Antidepressant treatments were performed both in MS and in n-MS rats and the results were compared with FSL rats that were not treated and not separated from mothers.

### 2.4. Neuronal Cultures

Hippocampal and cortical cultures were obtained from embryonic day 16.5 mice as previously described [47]. Procedures were approved by the Body for the Protection of Animals at the University of Trento and National Agency (793/2016-PR). Briefly, brains were collected in dissection media (HBSS 1X, 6 mM MgSO_4_, 10 mM HEPES pH 7.4, 10 mg/mL gentamycin). Primary cultures were cultivated at a density of 750–1000 cells/mm^2^ in neuronal complete medium (Neurobasal 1X, B27 supplement 1X, 0.5 mM l-glutamine, 10 mg/mL gentamycin). All culture reagents were purchased from ThermoFischer Scientific, Rodano, Italy. Cells were treated every second day starting on day in vitro (DIV) 5 until DIV14 either with ES at 5 µM dissolved in DMSO or NT at 1 µM dissolved in water; control groups received the respective vehicle. Antidepressants were purchased from Sigma Aldrich (Merck Life Science S.r.l., Milano, Italy).

### 2.5. RNA Extraction and cDNA Synthesis

For the in vivo study, rats were sacrificed by decapitation and brain regions were rapidly dissected, treated with RNAlater (Invitrogen, Thermofisher Scientific, Waltham, MA, USA) and stored at –80 °C. Total RNA was extracted with the Aurum total RNA fatty and fibrous tissue kit (Bio-Rad, Hercules, CA, USA) and quantified by absorbance in a NanoDrop 2000c UV-Vis spectrophotometer (ThermoFisher Scientific). cDNA was synthesised with the iScript Advanced cDNA synthesis Kit (Bio-Rad) following manufacturer’s instructions. In the in vitro experiments, total RNA was extracted with the Total RNA Purification kit (NORGEN Biotek, Thorold, ON, Canada) and quantified by absorbance in a NanoDrop 2000c UV-Vis spectrophotometer (ThermoFisher Scientific). cDNA was synthesised with the All-in-One Cdna Synthesis SuperMix (Bimake, Houston, TX, USA) following manufacturer’s instructions.

### 2.6. qPCR

Gene expression in rat hypothalamic samples was quantified by qPCR in real-time PCR reactions with Sybr Green technology in a 7900HT Fast Real-Time PCR System (Applied Biosystems, Thermofisher Scientific). Thirty nanograms cDNA were used in Sso Advanced Universal SYBR Green Supermix (Bio-Rad) at the following conditions: stage 1: 95 °C, 20 s; stage 2: 40× (95 °C, 3 s; 60 °C, 30 s). The primers were as follows: Adam10 fw 5′ GTTAACCCGTGAGGAGGCGG 3′; Adam10 rev 5′ GGCACGCTGGTGTTTTTGGT 3′; Negr1 fw 5′ CCTGGACGCAGTGGACTGAT 3′; Negr1 rev 5′ TGCTCCTGTGTCACGTTGGT 3′; Lsamp fw 5′ CACCAGGGAACAGTCAGGCA 3′; Lsamp rev 5′ TTGTCGTCCTGTGGTGGCTT 3′; Fgfr2 fw 5′ CCGGCCCTCCTTCAGTTTAG 3′; Fgfr2 rev 5′ TTCAACATGCAGCGCAACTC 3′; Gapdh fw 5′ AGACAGCCGCATCTTCTTGT 3′; Gapdh rev 5′ CTTGCCGTGGGTAGAGTCAT 3′. Primers were purchased from Eurofins, Vimodrone, Italy. The quantification was carried out by the Delta-Delta Ct method [48] by normalising to the reference gene Gapdh. A dissociation curve was built in the 60–95 °C range to confirm the specificity of the amplification product.

Gene expression in primary cultures was quantified by qPCR in real-time PCR reactions with Sybr Green technology in a CFX96 Touch Real-Time PCR Detection System (Bio-Rad). Five nanograms cDNA were used in iTaq Universal SYBR Green Supermix (Bio-Rad) at the following conditions: stage 1: 95 °C, 5 min; stage 2: 39× (95 °C, 15 s; 60 °C, 30 s). The primers were as follows: mNegr1 fw: GCGCTTGTTGCTCGAACCAG; mNegr1 rev: GATGCTCCATCTTCCAAGTAACAC; mLsamp fw: GGACCCTCGGGTTGAGCTG; mLsamp rev: CACAGTGACATCCGAGGAGATG; mAdam10 fw: GCAATTACATCATGTATGCAAGAG; mAdam10 rev: CTTCCCCTTGTTCCACCATC; mFgfr2 fw: CGCCTGTGAGAGAGAAGGAG; mFgfr2 rev: CTTCCCCTTGTTCCACCATC; mACTB1 fw: CAACGGCTCCGGCATGTG; mACTB1 rev: CTCTTGCTCTGGGCCTCG; mGAPDH fw: GAGAGTGTTTCCTCGTCCCG; mGAPDH rev: ACTGTGCCGTTGAATTTGCC. Primers were purchased from Metabion International AG. The quantification of the genes’ relative expressions was carried out by the Delta-Delta Ct method [48] by normalising to the reference genes Gapdh and B-Actin. A dissociation curve was built in the 60–95 °C range to confirm the specificity of the amplification product.

### 2.7. Transcriptional Analysis

Large-scale gene expression data on the cortex and the hippocampus were obtained from a dataset generated in our previous experiments carried out on FSL rats treated with antidepressants, which followed the same experimental design [38]. Briefly, the standard Affymetrix One-Cycle Eukaryotic Target Labelling Assay protocol was used to generate cRNA probes that were subsequently hybridised to Affymetrix Rat Genome 230 2.0 GeneChips (http://media.affymetrix.com/support/technical/datasheets/rat230_2_datasheet.pdf), following manufacturer’s guidelines (Affymetrix, Santa Clara, CA, USA) [38].

Additional data were extracted from the GEO dataset GSE43261 [49]. This dataset consisted of gene expression data on C57BL/6Ntac male mice treated for 3 weeks with 18 mg/kg/day fluoxetine (*n* = 11) or vehicle (*n* = 8) after 4 weeks of corticosterone (35 µg/mL/day) [43,49]. The processed version of the dataset was downloaded from GEO and used without further modifications. We extracted the values for the probes corresponding to *Adam10*, *Fgfr2*, *Lsamp*, and *Negr1*; since multiple probes were available for some of the genes of interest, for each sample we took the maximum value across the probes corresponding to the same gene as representative expression value for that gene.

### 2.8. Statistical Analysis

Relative gene expression was calculated by the Delta-Delta Ct method [48]; the mRNA level of each sample was expressed as a function of the average Delta Ct of the n-MS FSL group or the vehicle-treated group. Gene expression data were analysed using either 1-way or 2-way ANOVA approaches with stress (nMS and MS) and treatment (vehicle and antidepressant) as the factors of interest. When needed, an additional blocking factor plate was also included in the model to account for any plate-to plate variability as samples were analysed in different plates using a complete block design [50]. In all cases, the ANOVA analysis was followed by planned comparisons of the predicted means to compare the mean of the vehicle treated group to the mean of the antidepressant group.

The analyses were performed using InVivoStat software [51]. Data were log-transformed where appropriate to stabilise the variance and satisfy the parametric assumptions. A value of *p* < 0.05 was considered to be statistically significant.

## 3. Results

### 3.1. Antidepressant Treatments Alter Negr1-Fgfr2 Pathway Expression in Hypothalamus of FSL Rats

We aimed to investigate whether antidepressant treatment affected the expression of *Negr1* and other genes belonging to the same pathway in the hypothalamus, a brain region involved both in the regulation of feeding behaviour and in neuro-vegetative symptoms of MDD. We adopted a gene x environment MDD model to gauge if these factors provided different contributions, as we successfully accomplished in previous studies [41,42]. We used the Flinders strain because this model is able to reproduce behavioural, neurobiological, and molecular alterations observed in human depressed patients [34]. In addition, we subjected rats belonging to the FSL strain to the experience of maternal separation to model the early-life stress exposure experience that is deemed to be a causative factor for triggering MDD. Therefore, we aimed to compare the effect of antidepressant treatments in the susceptible strain subjected to early life stress to non-treated, non-separated animals. To reach this objective, chronic treatments with the serotonin-selective reuptake inhibitor escitalopram (ES) or the tricyclic antidepressant nortriptyline (NT) were carried out in parallel groups of both FSL rats exposed to post-natal maternal separation (MS) and in FSL controls that remained in close contact with their mothers (n-MS).

**Figure 1:**
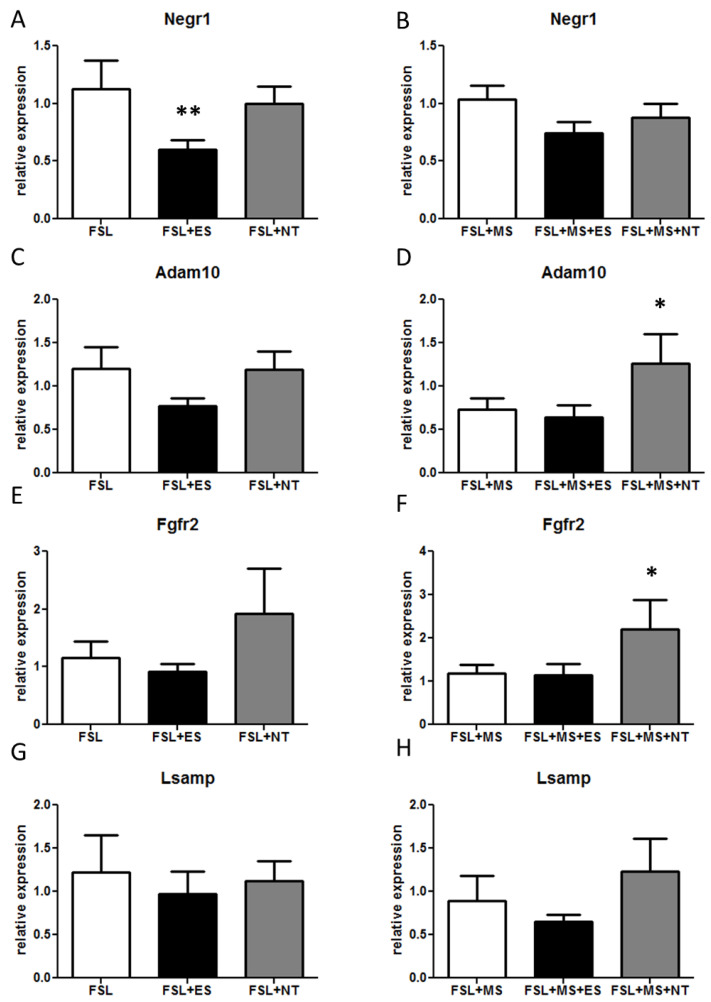
mRNA levels of genes belonging to the *Negr1-Fgfr2* pathway in the hypothalamus after ES or NT or vehicle administration. FSL rats were either left undisturbed in their home cage (n-MS, **A**,**C**,**E**,**G**) or exposed to MS (**B**,**D**,**F**,**H**) as described in the Methods section. (**A**,**B**) *Negr1* expression; (**C**,**D**) *Adam10* expression; (**E**,**F**) *Fgfr2* expression; and (**G**,**H**) *Lsamp* expression. * *p* < 0.05, ** *p* < 0.01 in the planned comparison vs. vehicle; *n* = 6/group.

In qPCR experiments, *Negr1* analysis showed a significant effect of treatment (F_2,20_ = 5.66, *p* = 0.011): In n-MS rats, *Negr1* expression was significantly reduced by 45% after chronic ES treatment (*p* = 0.0067; Figure 1A), whereas NT did not induce any change (Figure 1A).

In MS FSL no alterations were observed after antidepressant treatments (Figure 1B).

Similarly a treatment effect was detected in *Adam10* (F_2,25_ = 5.04, *p* = 0.014); in nMS rats *Adam10* expression showed a trend towards reduction in ES treated rats, although it did not reach statistical significance (*p* = 0.057, Figure 1C), while NT did not affect its mRNA levels. In contrast, NT treatment significantly increased *Adam10* by 70% (*p* = 0.030, Figure 1D) in MS rats, with no effect exerted by ES (Figure 1D).

Moreover, data showed that *Fgfr2* levels were also altered by the treatment (F_2,25_ = 9.45, *p* = 0.0009). *Fgfr2* was specifically increased by NT, whereas ES did not alter its expression (Figure 1E,F). Indeed, after NT administration *Fgfr2* expression was higher both in MS rats (*p* = 0.014, Figure 1E) and in n-MS animals (*p* = 0.063, Figure 1F). ES did not modify *Fgfr2*.

*Lsamp* RNA levels were not affected by antidepressant treatments in any condition (Figure 1G,H).

### 3.2. Antidepressant Treatments Induce Selective Alterations in Corticolimbic Regions of FSL Rats

FSL rats subjected, or not, to MS were treated with ES or NT and a global transcriptomic analysis was carried out in the hippocampus and prefrontal cortex. The experimental design was the same, with FSL rats subjected to MS and receiving chronic antidepressant treatment. Parallel groups of n-MS FLS rats which were not treated with antidepressants formed the control groups. We extracted the expression results from probes specific for genes belonging to the *Negr1-Fgfr2* pathway and compared them in all experimental groups.

**Figure 2:**
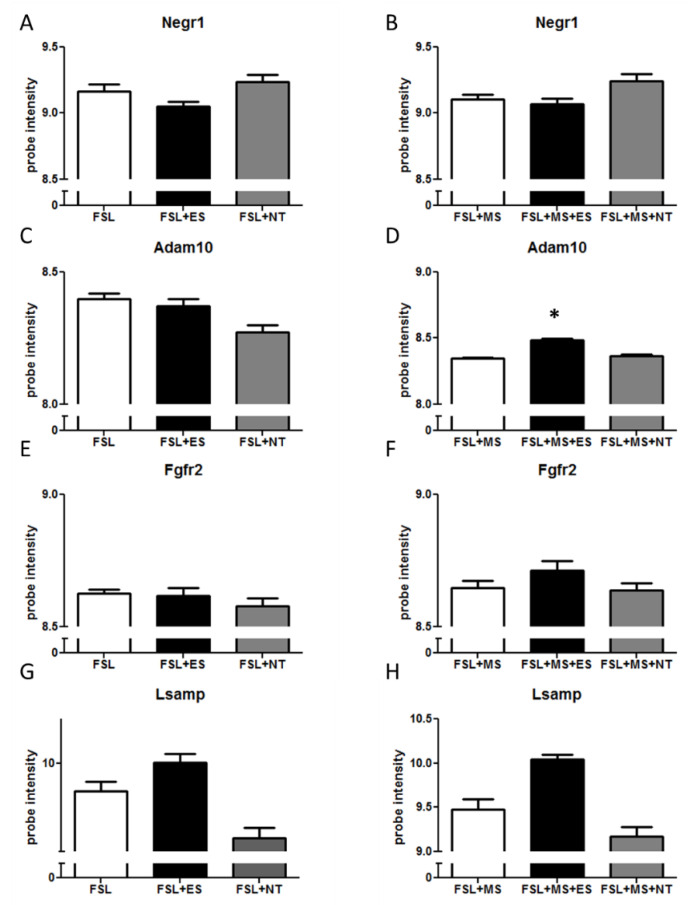
mRNA levels of genes belonging to the *Negr1-Fgfr2* pathway in the hippocampus after ES or NT or vehicle administration. FSL rats were either left undisturbed in their home cage (n-MS, **A**,**C**,**E**,**G**) or exposed to MS (**B**,**D**,**F**,**H**) as described in the Methods section. (**A**,**B**) *Negr1* expression; (**C**,**D**) *Adam10* expression; (**E**,**F**) *Fgfr2* expression; and (**G**,**H**) *Lsamp* expression. * *p* < 0.05 in the planned comparison vs. vehicle; *n* = 8–10.

In the hippocampus, a stress x treatment effect was detected (F_2,67_ = 4.58, *p* = 0.014). *Adam10* expression decreased after NT administration in n-MS rats (*p* = 0.071, Figure 2C), whereas ES treatment augmented it in the MS group (*p* = 0.017, Figure 2D).

The other tested genes were not modified by antidepressant treatments in any condition (Figure 2A,B,E,H).

**Figure 3:**
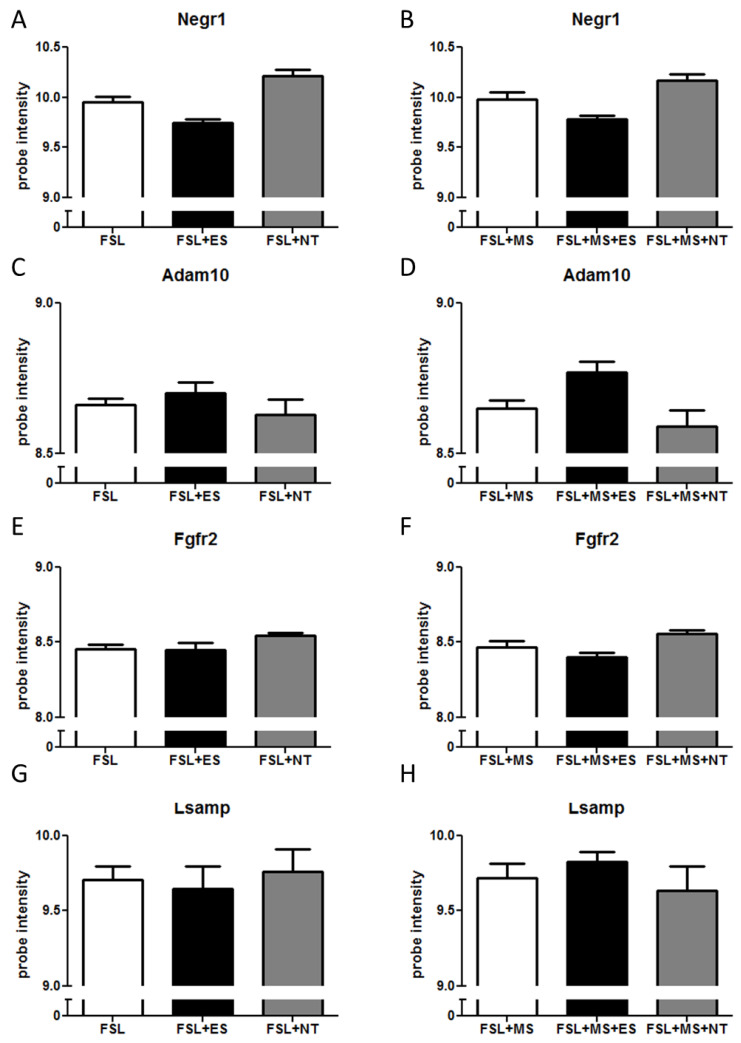
mRNA levels of genes belonging to the *Negr1-Fgfr2* pathway in the pre-frontal cortex after ES or NT or vehicle administration. FSL rats were either left undisturbed in their home cage (n-MS, **A**,**C**,**E**,**G**) or exposed to MS (**B**,**D**,**F**,**H**) as described in the Methods section. (**A**,**B**) *Negr1* expression; (**C**,**D**) *Adam10* expression; (**E**,**F**) *Fgfr2* expression; and (**G**,**H**) *Lsamp* expression. *n* = 8–10.

In the pre-frontal cortex, no gene belonging to the *Negr1-Fgfr2* pathway showed any treatment-induced alterations (Figure 3).

### 3.3. Negr1-Fgfr2 Pathway Expression in the Behavioural Response to Fluoxetine in the Chronic Corticosterone Mice Model

Subsequently, we addressed the question of whether the response we observed to antidepressant treatments in the *Negr1* pathway was associated with behavioural antidepressant response. Available evidence showed that a mouse model in which depressive-like behaviours were induced by chronic corticosterone treatment corresponded to the requirements [49]. In this model, long-term exposure to low doses (35 µg/day for four weeks) of corticosterone induces an anxiety and depressive-like state which is amenable to being reversed by chronic antidepressant treatment [43]. In particular, three-week treatment with the serotonin-selective reuptake inhibitor fluoxetine reversed depressive-like behaviour in the forced swim test in most mice, whereas a sub-set displayed resistance [49]. We therefore aimed to compare the expression of *Negr1*-related genes between vehicle and fluoxetine-treated mice, including a comparison between fluoxetine-sensitive and resistant mice. To reach this objective, we extracted and analysed the data regarding the expression of *Negr1*, *Adam10*, *Fgfr2*, and *Lsamp* from an open-access dataset which was generated by performing a large-scale transcriptomic analysis on this model, including both fluoxetine-sensitive and fluoxetine-resistant mice brains [49].

**Figure 4:**
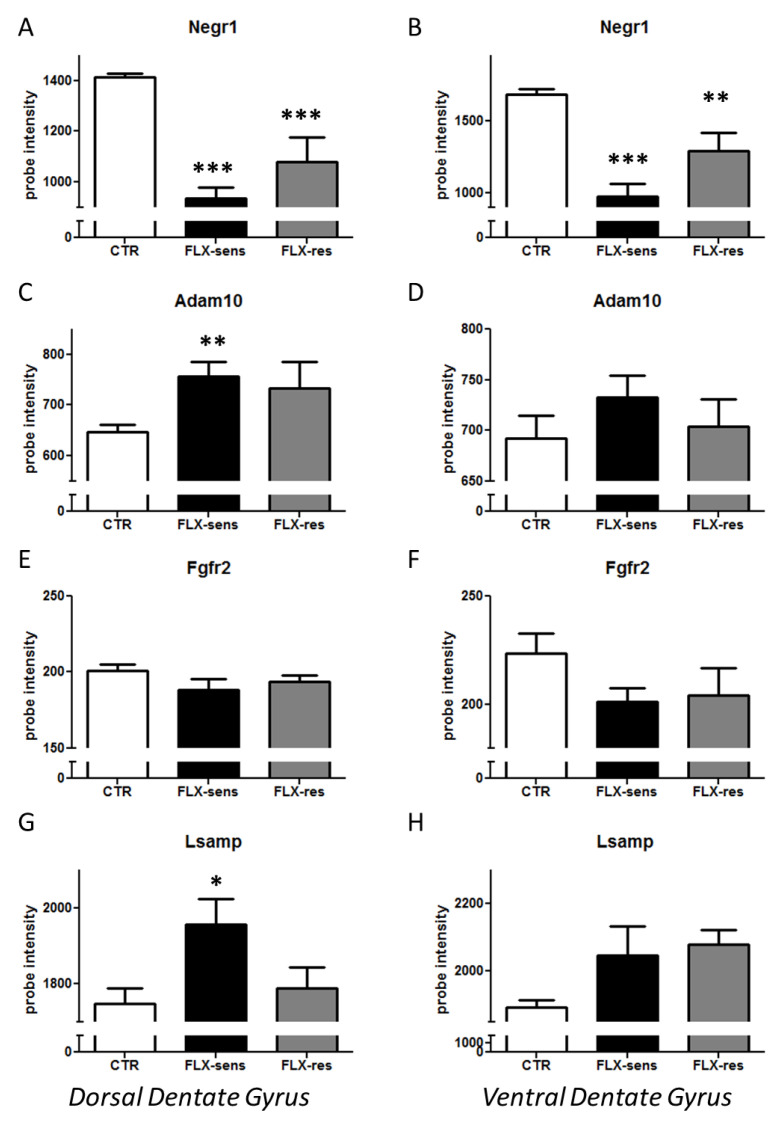
mRNA levels of genes belonging to the *Negr1-Fgfr2* pathway in the dorsal dentate gyrus (**A**,**C**,**E**,**G**) or ventral dentate gyrus (**B**,**D**,**F**,**H**) of the hippocampus after fluoxetine or vehicle administration. Groups showing fluoxetine behavioural sensitivity or resistance are shown as black or grey columns, respectively. (**A**,**B**) *Negr1* expression; (**C**,**D**) *Adam10* expression; (**E**,**F**) *Fgfr2* expression; and (**G**,**H**) *Lsamp* expression. * *p* < 0.05, ** *p* < 0.01, *** *p* < 0.001 in the planned comparison vs. vehicle. FLX: fluoxetine. *n* = 4–8.

In the dorsal dentate gyrus, fluoxetine treatment induced a dramatic decrease in *Negr1* mRNA (F_2,16_ = 35.12, *p* < 0.0001) in both sensitive and resistant mice (*p* < 0.0001 and *p* = 0.0002, respectively), but the amount appeared larger in the sensitive group (Figure 4A).

We also discovered that *Adam10* expression was increased (F_2,16_ = 5.23, *p* = 0.018) in both fluoxetine-sensitive and resistant mice, but the signal appeared stronger in sensitive mice (*p* = 0.0067 and *p* = 0.057, respectively, Figure 4B). In contrast, *Lsamp* levels (F_2,16_ = 4.39, *p* = 0.030) were specifically augmented only in the antidepressant-sensitive group (*p* = 0.011, Figure 4G).

In the ventral dentate gyrus, like in the dorsal region, *Negr1* expression was significantly reduced (F_2,16_ = 22.51, *p* < 0.0001) in both antidepressant-treated groups, with both confidence and amount larger in sensitive animals (*p* < 0.0001 in sensitive mice, *p* = 0.0065 in resistant mice, Figure 4B). Again, similarly to dorsal dentate gyrus, *Lsamp* levels (F_1,16_ = 2.84, *p* = 0.087) showed a trend towards increase in both fluoxetine-sensitive and resistant mice (*p* = 0.068 and *p* = 0.060, respectively; Figure 4H).

### 3.4. Alterations of the Negr1-Fgfr2 Pathway in Primary Neuronal Cultures Treated with Antidepressants

**Figure 5:**
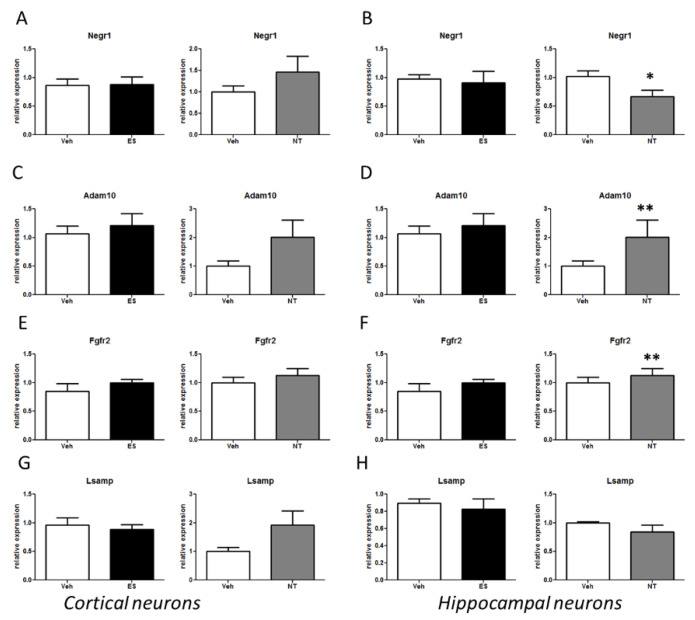
mRNA levels of genes belonging to the *Negr1-Fgfr2* pathway in cortical and hippocampal cultures after ES or NT or vehicle administration. Cultures were treated every second day from DIV5 to DIV14 either with escitalopram (ES, 5 µM) and vehicle (DMSO) or nortriptyline (NT, 1 µM) and vehicle (water). (**A**,**B**) *Negr1* expression in hippocampal or cortical neurons; (**C**,**D**) *Adam10* expression; (**E**,**F**) *Fgfr2* expression; and (**G**,**H**) *Lsamp* expression. Data are expressed as mean ± S.E. * *p* < 0.05, ** *p* < 0.01 Student’s T-test, *n* = 9–10.

The data obtained in two different rodent models of MDD showed that chronic antidepressant treatment alters gene expression in the *Negr1-Fgfr2* pathway. Next, we aimed to investigate whether the alterations in the *Negr1-Fgfr2* pathway that we detected in rodent MDD models were directly cell-autonomous or network-based. Several findings show that anti-depressant treatment induces cell-specific molecular alterations. Thus, we analysed the impact of chronic treatment with ES or NT in two well-established in vitro neuronal models, namely primary cortical and hippocampal cultures. We treated cells every second day starting at DIV5 either with ES (5 µM) and vehicle (DMSO) or NT (1 µM) and vehicle (water). At DIV14, we extracted the mRNA and analysed by qPCR the relative expression of *Negr1*, *Lsamp*, *Adam10*, and *Fgfr2*. Neither ES nor NT treatments had an overt effect on *Negr1* and its related pathway in the cortical cultures (Figure 5). Instead, chronic treatment with NT induced a significant decrease of *Negr1* and increase of *Adam10* and *Fgfr2* expression in the hippocampal cultures (*Negr1 p* < 0.05; *Adam10 p* < 0.01; *Fgfr2 p* < 0.01; Figure 5B,D,F).

## 4. Discussion

The aim of this study was to investigate if the *Negr1-Fgfr2* pathway was modulated by antidepressant treatment, since this gene has been genetically associated to both MDD and obesity, two co-occurring conditions believed to share common neurobiological alterations [52]. We discovered that members of this pathway were influenced by antidepressant treatment in animal models of disease and in primary neurons.

We found that *Negr1*, a cell adhesion molecule expressed in neurons, was significantly altered by chronic antidepressant treatment in animal models of MDD. In the hypothalamus, ES treatment reduced *Negr1* expression in FSL MS rats; similarly, fluoxetine administration decreased *Negr1* levels in hippocampal dentate gyrus, and more significantly in fluoxetine sensitive animals (Table 1).

NT did not significantly modulate *Negr1* in treated animals, but it decreased *Negr1* levels in hippocampal primary neurons (Table 1). *Adam10*, a metallopeptidase which cleaves Negr1, thus releasing its soluble portion [25], was increased by NT administration in the hypothalamus and by ES in the hippocampus of MS FSL rats, as well by fluoxetine in the dorsal dentate gyrus of fluoxetine sensitive mice (Table 1). In parallel, NT increased *Adam10* expression in hippocampal cultures (Table 1). Fgfr2 can be an effector of Negr1 soluble portion to influence neurite outgrowth [25]. We discovered that its expression was increased by NT treatment in the hypothalamus of FSL rats, as well as in hippocampal neurons (Table 1), while no changes were detected with other antidepressants or in other brain regions. *Lsamp*, another member of the immunoglobulin-related cell-adhesion molecules, was not significantly affected by ES or NT treatment in FSL rats or in cultures. However, it was found to be increased in the dentate gyrus after fluoxetine treatment in the mice model of depression (Table 1). This finding is in agreement with data collected in *Lsamp*^–/–^ mice, which showed that this adhesion molecule displayed a modulatory role in 5-HT signalling [20].

Evidence from cognitive neuroscience indicates that MDD originates from dysfunctional neuronal connectivity in various brain regions that begins in adolescence before the brain approaches its adult anatomical state [53]. Brain MR imaging studies in MDD based on different approaches found evidence of altered volume or aberrant activation in frontal regions, in the hippocampus, and in the thalamic regions [45,54,55]. However, the plethora of brain regions displaying aberrant neural activity along with the inconsistency of these findings across studies has led researchers to move beyond a single-brain-region model of illness for MDD, and to hypothesise that MDD could originate from altered connectivity between brain regions [56]. The first evidence from Mayberg and colleagues [57] who described a relationship between the metabolic alteration in the prefrontal cortex and in the limbic regions resulted in the hypothesis that a corticolimbic connectivity abnormality may underlie MDD. These hypotheses have been corroborated by studies showing abnormalities of coupling between brain region activations in response to emotional stimuli, decreased anterior cingulate connectivity with the amygdala, thalamus, and striatum, and altered connectivity of the corticolimbic or intracortical connectivity [58]. Moreover, an interplay between brain connectivity and pharmacological treatment has been demonstrated for several psychiatric illnesses, including MDD, where associations between response to antidepressant medications and increased frontolimbic functional connectivity have been reported [59]. In summary, connectivity abnormalities in corticolimbic networks offer a more comprehensive, robust, and integrated model for MDD.

In line with evidence supporting the notion that the development of MDD is induced by alterations of functional neural circuits underlying mood regulation [60], chronic antidepressant administration increases synaptic plasticity at numerous levels and this ability is considered central for therapeutic efficacy [61,62,63]. Indeed, the rapid response induced by faster-acting antidepressants is believed to rely on their ability to induce changes in synaptic function and plasticity [60,64].

Notwithstanding the difference in specific brain areas, overall the present findings show that antidepressant treatments were able to influence the *Negr1-Fgfr2* pathway and support the hypothesis that Negr1-mediated modulation of neuronal plasticity is activated by antidepressant treatment. We and others have demonstrated that Negr1 and Lsamp modulate neurite arborisation and the formation of neuronal circuits by stimulating specific intracellular signalling after metalloproteinase-dependent shedding [14,24,25,65,66,67]. Our findings are in keeping with the hypothesis that chronic treatment with monoaminergic-modulating antidepressants is able to influence synaptic plasticity in brain regions involved in the regulation of mood and affect and that IgLON protein pathways play a role in the process. At the cellular level, post-mortem studies have described a robust reduction in synapse number in the PFC of MDD patients [68]. These findings have been complemented and expanded by observations in several preclinical models [69,70].

The *Adam10-Negr1-Fgfr2* pathway is known to influence spine plasticity. Adam10 regulates dendritic spine morphology and glutamate receptor composition through substrate cleavage. Our previous studies demonstrated that Negr1 is a membrane-bound protein that can be released into the extracellular environment upon Adam10 shedding [25]. Membrane-bound Negr1 promotes the intracellular trafficking of Fgfr2 thus regulating the formation of the upper layers in the cortical areas [71]. Soluble Negr1 regulates through Fgfr2 synapse formation of hippocampal neurons, neurite outgrowth, and dendritic spine plasticity [24,25].

Interestingly, antidepressants influence the *Adam10-Negr1-Fgfr2* pathway. Preliminary evidence suggests that serotoninergic antidepressants can affect the expression of *Adam10* in platelets [72] and *Negr1* in rat brains [73]. Taken together, these lines of evidence indicate that *Adam10-Negr1-Fgfr2* pathway biology is tightly linked to the molecular events underlying MDD therapeutic interventions.

We discovered that different responses were elicited in MS with respect to n-MS FSL rats. Our previous studies investigated the neurochemical, behavioural, and molecular responses of this rodent model of MDD to antidepressant treatments [39,40,41,42,74,75,76,77]. Our previous findings suggested an association between MS FSL animal behaviours and treatment-resistant MDD [38]. Following this hypothesis, we can speculate that molecular alterations specifically observed in n-MS groups can be more confidently associated with the neurobiological changes involved in antidepressant efficacy. In agreement with this notion, the results observed in fluoxetine-sensitive rats show that in several instances the direction of change is similar, whereas the amount is greater in fluoxetine-sensitive groups. This fact suggests that an adequate stimulus needs to be present to allow efficient activation of neuronal plasticity.

Previous studies oriented at identifying genetic variants able to differentially predict outcome of treatment with antidepressants in depressed patients showed that no association reached the genome-wide level of significance [78]. Although not significant after correction for multiple testing, one of the most interesting results showed that a single nucleotide polymorphism (SNP) in the *Negr1* gene was associated with the differential response to serotonergic and noradrenergic antidepressants [78]. This finding provides further support to the present data which revealed antidepressant-induced *Negr1* modulation and allows speculating that this expression change is requested to trigger a neuronal plasticity response necessary for therapeutic efficacy.

In addition to the activity exerted by modulation of monoaminergic circuits, our data showed that tricyclic anti-depressants directly tune *Adam10-Negr1-Fgfr2* mRNA expression levels. We found that NT significantly reduced *Negr1* and increased *Adam10* and *Fgfr2* expression in hippocampal cultures but not in cortical cultures. This outcome may reflect the different battery of serotonin (5HT) and norepinephrine receptors expressed in the different neuronal population. Alpha and beta adrenergic receptors are expressed at both pre- and postsynaptic sites in the hippocampus [79] and in the cortex [80] where they participate in LTP and LTD phenomena. Cortical neurons express different 5HT receptors, with 5HTA2 and 5HT1A receptors expressed at the highest levels [81]. Instead almost all pre- and postsynaptic serotonin receptors have been identified in the hippocampus [82].

Both serotonergic and adrenergic receptors impinge on MAPK/ERK activation and thus control gene expression. Therefore, antidepressant treatment might influence *Negr1*, *Adam10*, and *Fgfr2* gene expression in parallel via ERK. However, it has been demonstrated that antidepressants activate the Fgfr signalling cascade [83]. Thus, it is tempting to speculate that Fgfr2 activation is the first event along the molecular cascade underlying antidepressant therapeutic effect. Our previous data suggest that Negr1 plays a major role within this cascade.

The question now is to understand how altered levels of *Negr1* may correlate with MDD pathology and therapy. Given that Negr1 acts as an Fgfr2 agonist and that Fgfr2 signalling is pivotal within antidepressant therapeutic effect, the most convenient model would imagine that low Negr1 levels are pathological. Instead, genetic and experimental data suggest just the opposite. One hypothesis is that the chronic increase of Negr1 level may down-regulate Fgfr2 signalling while antidepressants succeed in restoring its physiological activity.

## Figures and Tables

**Table 1:** Overview of the direction of gene expression modulation exerted by chronic antidepressant treatments for genes belonging to the *Negr1-Fgfr2* pathway. Cort: corticosterone; Flx: fluoxetine; sens: fluoxetine-sensitive; res: fluoxetine resistant; Hip DG: hippocampal dentate gyrus.

Genes	Flinders Sensitive Rats (ES, NT)			Cort-Treated Mice (FLX)		Primary Neurons (ES, NT)	
	Hippocampus	Hypothalamus	Cortex	Hip DG (dorsal)	Hip DG (ventral)	Hippocampus	Cortex
*Negr1*	-	↓↓ nMS ES	-	↓↓↓sens ↓↓↓res	↓↓↓sens ↓↓res	↓ NT	-
*Adam10*	↑ MS ES	↑ MS NT	-	↑↑ sens	-	↑↑ NT	-
*Fgfr2*	-	↑ MS NT	-	-	-	↑↑ NT	-
*Lsamp*	-	-	-	↑ sens	-	-	-
